# Occurrence and Genetic Variation of *Monolepta hieroglyphica* (Motschulsky, 1858) (Coleoptera: Chrysomelidae), a Newly Emerging Pest, Among Hosts in Northeast China

**DOI:** 10.3390/insects16060605

**Published:** 2025-06-08

**Authors:** Wei Sun, Xiuhua Zhang, Jiachun Zhou, Yuebo Gao

**Affiliations:** Northeast Agricultural Research Center of China, Institute of Plant Protection, Jilin Academy of Agricultural Sciences, Gongzhuling 136100, China; swswsw1221@sina.com (W.S.); hshs922922@163.com (X.Z.); pxzkn1130@163.com (J.Z.)

**Keywords:** leaf beetle, host population, spatial dynamics, dispersal, molecular marker, genetic diversity

## Abstract

The northeast region of China plays a crucial role in crop production. The leaf beetle *Monolepta hieroglyphica* (Motschulsky, 1858) (Coleoptera: Chrysomelidae) has emerged as a potential threat to food security in the region. With a wide distribution spanning Asia and Russia, this beetle affects various crops. However, limited information is available regarding its occurrence patterns and genetic diversity among major crops in the region. Based on systematic observations across various hosts, coupled with genetic variation analysis using mitochondrial DNA markers, the main results were as follows. Leaf beetle occurrence varied among hosts, peaking from late July to mid-August, with maize and soybean fields exhibiting higher infestation rates compared with other crops. Notably, late-cultivated maize fields harbored the highest beetle numbers due to the species’ preference for young leaves. The host transfer trajectory may have originated in soybean and weeds, with subsequent alternation between host plants and other crops, before the final migration to cabbage and late-cultivated maize fields. Genetic analysis revealed nine COI haplotypes, four COII haplotypes, eleven Cytb haplotypes, and twenty-one combined haplotypes. No clear relationship existed between genetic diversity and occurrence, and no distinct host-based genetic patterns emerged from neighbor-joining tree and haplotype network analyses. High gene flow rates were observed, likely contributing to decreased genetic variation. An analysis of molecular variance results indicated major genetic variation within populations, although genetic distance and haplotype distribution indicated divergence among host populations. These results provide foundational data for developing effective *M. hieroglyphica* pest management strategies.

## Simple Summary

Northeastern China is recognized as a crucial grain-producing region, but food security is severely affected by diverse pests. Due to changes in climate, cultivation patterns, and crop distribution, the leaf beetle *Monolepta hieroglyphica* (Motschulsky, 1858) (Coleoptera: Chrysomelidae) has emerged as a destructive pest. However, its occurrence across different hosts remains poorly understood. This study analyzed the pest’s occurrence patterns and genetic diversity through systematic observation and mitochondrial DNA markers. These findings are essential for developing effective pest control strategies in the region.

## 1. Introduction

Northeastern China, including Eastern Inner Mongolia, Jilin, Liaoning, and Heilongjiang Provinces, is regarded as the country’s largest grain production base. With large plain topography, this region benefits from an April–October growing season, and harsh winters limit insect activity. Major crops include maize, soybean, and rice, with other crops such as sunflower, wheat, millet, peanut, and sorghum cultivated in certain areas. The region experiences substantial agricultural losses due to various pests, including the oriental armyworm *Mythimna separata* (Walker) (Lepidoptera: Noctuidae) and aphids [1,2]. However, due to changes in climate, cultivation patterns, and crop allocation, the leaf beetle *Monolepta hieroglyphica* (Motschulsky, 1858) (Coleoptera: Chrysomelidae) has emerged as a new threat to food security, particularly affecting maize and soybean crops [3,4].

This pest is widely distributed across East Asia, Southeast Asia, and Russia [5]. In China, this species exhibits a broad provincial distribution, with overwintering occurring in the egg stage [3]. This leaf beetle is a polyphagous pest, feeding on a wide variety of crop and weed species, with larvae and adults directly inflicting crop damage [6]. The larvae are an underground pest of crop plants, whereas the adults damage leaves, flowers, filaments, pollen, floral organs, clusters, and grains, and may negatively affect pollination [7]. Adults possess wings, allowing some mobility, including short-distance dispersal (2–5 m) [6].

The economic impact of the pest has prompted extensive research into its occurrence patterns, phylogenetics, insecticidal mechanisms, drip irrigation control, and biological control [4,8,9,10]. Studies on *M. hieroglyphica* genetics have primarily examined its complete mitochondrial genome, molecular systematics, and molecular markers [3,11,12,13,14]. However, little attention has been paid to the pest’s occurrence among different hosts, resulting in limited understanding of host transfer migration. Furthermore, clear differentiation exists among *M. hieroglyphica* geographic populations in northern China [13], indicating population-level variation. Similarly, morphology and biological differences exist among host populations, such as variations in adult size related to emergence periods [7] and discrepancies in life history and reproduction [15]. However, it remains unclear whether these differences are due to host specialization. This scientific question warrants further investigation.

This study explores the spatial dynamics and genetic variations of the leaf beetle across different crops in Northeast China. Understanding occurrence patterns and genetic diversity is pivotal for devising effective pest management strategies. A key aspect of this study lies in the experimental site selection, which included all host plant species within a relatively small area, eliminating topographical, temporal, geographic, and climatic differences that could impede the pest’s dispersal. Another innovative aspect involved systemic field surveys combined with molecular markers, proving valuable for studying host genetic variation. The study’s findings provide unique regional insights into pest management.

## 2. Materials and Methods

### 2.1. Population Dynamics Analyses

Field surveys were conducted in Gongzhuling (43°32′09″ N, 124°49′28″ E), situated in the central agricultural plain of Jilin Province, during the periods of June to October in 2022 and 2023. In total, 11 host fields were established, including the major crops in Northeast China. The experimental site and sampling details are provided in Table 1. Weed species primarily belonged to the grass family. Planting in most host fields occurred during April and May. Cabbage seed planting was performed on 12 August 2022 and 2 August 2023, aligning with general practices in the local area. Late-cultivated maize [maize (L)] seeds were planted on 2 July 2022 and 30 June 2023.

Field observations were conducted using sweep sampling, visual observations, and yellow traps (Table 1). Sampling methods were adjusted for specific host fields based on their unique characteristics. Sweep sampling is a common method used to estimate the relative abundance of insect communities. Following O’Neill et al. [16] and Whipple et al. [17], 200 random sweeps with a 40 cm diameter sweep net were performed per field. Sweep sampling was used for low-density plant fields, such as weeds and soybean fields. For other host fields, visual observations were performed during random 200 m walking surveys in each field. Notably, field observations in maize (L) were conducted over 120 m from July to October 2022, and over 180 m from July to October 2023. In contrast, cabbage field observations covered 120 m from September to October 2022, and 150 m from August to October 2023. Yellow traps, which effectively record pests’ initial and last appearance, were used as supplementary tools. Two yellow traps (20 × 40 cm) were placed at the center of each field, with data recorded every 3 days June–October in 2022 and 2023.

### 2.2. Molecular Analyses

Mitochondrial DNA (mtDNA) serves as a valuable molecular marker for assessing population genetic diversity and variation [18,19,20,21,22,23]. Partial COI, COII, and Cytb fragments of mtDNA were selected for use and amplified using the following primer pairs (Table 2). To ensure consistency across sampling years, 10 host populations of *M. hieroglyphica* were collected in 2022 and 2023 from the aforementioned host fields and stored at −20 °C until processing. Table 1 presents the host population sample sizes used for molecular analysis. Among the 337 samples subjected to PCR amplification, differential gene conservation resulted in varying success rates: 299 samples amplified successfully for COI, 324 for COII, and 310 for Cytb. Owing to low occurrence, samples from wheat were not collected. To ensure accuracy, only samples from yellow traps in maize (L) fields were included in the statistical analysis, accounting for the relatively small sample size. Morphologically, identification was performed by Wei Sun using reference materials [24].

All the experimental procedures, including PCR design and sequencing, were conducted by Sangon Biotech (Shanghai) Co., Ltd. In total, 337 samples were used for genomic DNA extraction. Genomic DNA was extracted from a portion of *M. hieroglyphica* adult bodies using a genomic DNA purification kit (Sangon Biotech, Shanghai, China). PCR reaction mixtures contained 1 µL of DNA template, 2.5 µL of Taq buffer (with MgCl_2_), 1 µL of each primer, 1 µL of dNTP, and 0.2 µL of Taq DNA polymerase enzyme (Sangon Biotech, Shanghai, China) in a 25 µL volume with molecular-grade water. PCR cycling parameters were as follows: initial denaturation at 95 °C for 5 min; 10 cycles of denaturation at 94 °C for 30 s, annealing at 63 °C for 30 s (decreasing by 0.5 °C per cycle), extension at 72 °C for 30 s; 30 cycles of denaturation at 95 °C for 30 s, annealing at 58 °C for 30 s, extension at 72 °C for 30 s; and a final extension at 72 °C for 10 min. All PCR reactions were conducted using an ABI Veriti 96-Well system, and samples with successful PCR amplification were sequenced using the ABI 3730 XL (Applied Biosystems, Foster City, CA, USA).

### 2.3. Data Analyses

The data from observations/sweep sampling and yellow traps were combined for comprehensive analysis, aiming to provide more accurate and complementary information. Statistical analyses and visualizations were performed using Excel 2010. To provide a comprehensive understanding of genetic variation, COI, COII, and Cytb fragments were analyzed both individually and in combination. Sequence alignment, editing, and haplotype definition were performed using Chromas 1.62, DNAMAN V6, and EditSeq 5.01 software. Haplotypes were deposited in the NCBI Genbank database under accession numbers PP038011–PP038019 and PP056518–PP056532. Nucleotide composition, variable sites, transition/transversion ratios, and haplotype genetic distances were calculated using MEGA 4.0 [25]. A phylogenetic tree [neighbor-joining (NJ)] was constructed with the K-2-P model in MEGA 4.0. DnaSP 5 was used to analyze haplotype number (*H*), haplotype diversity (*Hd*), average number of nucleotide differences (*K*), nucleotide diversity (*Pi*), and gene flow estimates [26]. Haplotype networks were generated using Network 4.6.1.6 with median joining [27]. Analyses of molecular variance (AMOVA) and population genetic distance were performed using Arlequin 3.5.1.2 [28].

## 3. Results

### 3.1. Population Dynamics

**Figure 1:**
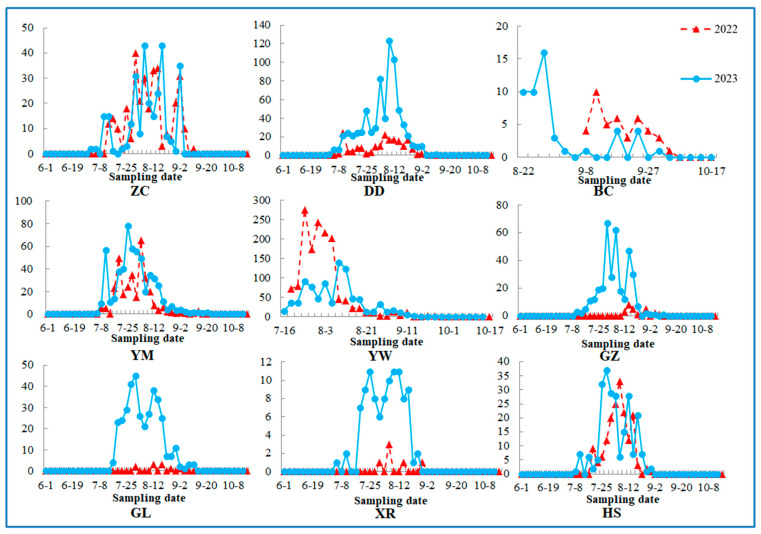
Population dynamics of the leaf beetle among host plant species in 2022 and 2023. The wheat field is excluded owing to low occurrence levels. ZC: weed; DD: soybean; BC: cabbage; YM: maize; YW: maize (L); GZ: millet; GL: sorghum; XR: sunflower; HS: peanut; XM: wheat. The same applies below.

The population dynamics of *M. hieroglyphica* based on the field-collected data are shown in Figure 1. During 2021 and 2022, in the millet field, leaf beetles first appeared on 10 July and last appeared on 11 September, with peak abundance observed from late July to late August. Similarly, in the sunflower field, the initial appearance occurred on 4 July, with the last sighting on 27 August and peak abundance from late July to mid-August. In the peanut field, leaf beetles first and last appeared on 8 July and 27 August, respectively, with peak abundance from late July to mid-August. In the sorghum field, the first sightings were on 17 July, with the last observation on 11 September and peak abundance from late July to late August. The wheat fields exhibited minimal activity, with only one individual observed. Regarding the maize field, leaf beetles first occurred on 2 July, last occurred on 1 October, and peaked from mid-July to mid-August. In the maize (L) field, the beetles were first observed on 16 July and last sighted on 23 September, with peak abundance occurring from late July to mid-August. The cabbage fields first showed the presence of leaf beetles on 22 August, with the last occurrence on 5 October. Regarding both soybean fields and weeds, the beetles initially appeared on 2 July and were last found on 11 September; however, peak abundance was from mid-July to late August in soybean and from mid-July to early September in weeds.

The field observation data from the rice field closely resembled those from other host populations. Given that the study area primarily consists of dry farmland with minimal rice cultivation (not representative of major rice production zones), rice field data were excluded from the statistical analysis to maintain research accuracy. Only molecular study samples were used. No individuals were detected after crop harvesting. After September, no samples were collected in the sunflower and peanut fields, likely due to their earlier harvest. Based on the above observation, the peak occurrence period occurred from late July to mid-August. Combined data from the two sampling years revealed that leaf beetle occurrence commenced earlier in maize, soybean, and weed hosts, whereas in later periods, the pest shifted to maize, maize (L), and cabbage fields.

**Figure 2:**
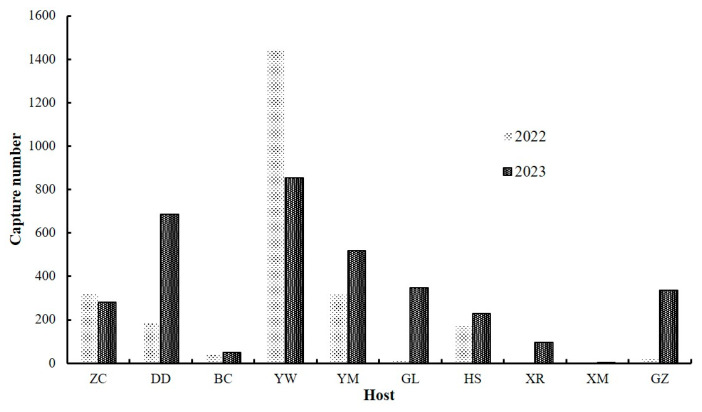
Occurrence levels of the leaf beetle among various hosts.

Despite differences in sampling methods, comparable numbers were obtained (Figure 2). Occurrence rates were relatively higher in 2023. Substantial numbers were observed in the maize (L) field, which made a major contribution to abundance in 2022. Distribution among hosts was more balanced in 2023. In a comprehensive analysis of two-year data, the maize (L) field showed the highest occurrence (2298 individuals) despite the reduced sampling period and distance. The maize (835) and soybean (870) fields exhibited relatively high numbers compared with other crops. The weed (600), sorghum (357), millet (361), and peanut (399) fields also contained numerous beetles, whereas the cabbage fields (92) showed lower numbers, partly attributed to the reduced period and distance. The sunflower fields (101) exhibited fewer individuals, and only one adult was captured in the wheat field.

### 3.2. Base Composition

The alignment of the COI sequences contained 615 bases with 607 conserved sites and eight single variable sites. The average nucleotide composition was as follows: A: 37.7%; T: 32.8%; C: 15.8%; and G: 13.7%. The transition/transversion ratio (R) was 7. The average A + T content was 70.5%. The alignment of COII sequences, containing 430 bases, had 427 conserved sites and three single variable sites. The average nucleotide composition was as follows: A: 34.9%; T: 41.6%; C: 12.8%; and G: 10.7%. The average A + T content was 76.5%. The alignment of Cytb sequences comprised 430 bases with 423 conserved sites. The sequence included two single variable sites and five parsimony-informative sites. The average nucleotide composition was as follows: A: 40.5%; T: 34.3%; C: 11.3%; and G: 13.9%. The transition/transversion ratio (R) was 12.6. The average A + T content was 74.8%.

The combined COI, COII and Cytb fragment, containing 1475 bases, had 1458 conserved sites and 17 single variable sites. The sequence included nine single variable sites and eight parsimony-informative sites. The average nucleotide composition was as follows: A: 37.7%; T: 35.8%; C: 13.7%; and G: 12.9%. The transition/transversion ratio (R) was 16.4. The average A + T content was 73.5%. The COI, COII, and Cytb fragments were identified with 100% confidence using previously submitted sequences from NCBI (accession nos. MW732714.1). No additions or deletions were observed. Substitutions were predominantly transitions, notably C-T patterns. The high A + T content was consistent with typical insect values.

### 3.3. Haplotypes

**Figure 3:**
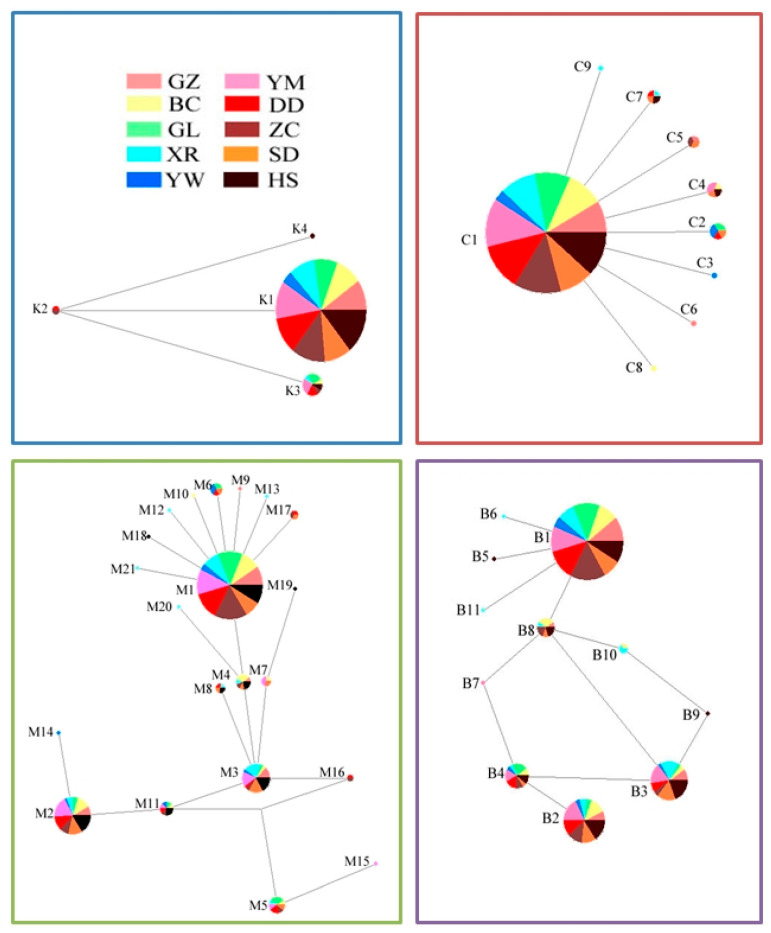
Leaf beetle networks were constructed based on COI, COII, Cytb, and the combined haplotypes. Each haplotype is represented by a circle, with the circle size proportional to the haplotype frequency. Different colors represent different host groups. C1–C9: mtDNA COI haplotypes 1–9. K1–K4: mtDNA COII haplotypes 1–4. B1–B11: mtDNA Cytb haplotypes 1–11. M1-M21: the combined haplotype 1–21. The same applies below.

The established network, reflecting haplotype frequencies and distributions (Figure 3), showed no evidence of host plant trends. From 299 individuals, nine unique mtDNA COI haplotypes (C1–C9, NCBI accession nos. PP038011-PP038019, Table S1) were identified. The haplotype content was 3% (9/299). Haplotype C1 was ubiquitous across host populations, constituting 92.64% of individuals. Haplotype C2, the second most frequent haplotype (2.01% of individuals), occurred in four host populations, as did haplotype C4 (1.67% of individuals). Notably, haplotype C4 was consistently present in the maize field across both sampling years. Haplotype C7 was observed in four host populations, representing 1.34% of individuals. The remaining infrequent haplotypes were distributed irregularly among different populations, with the infrequent haplotype C5 detected in soybean and weed populations. The mean genetic distance among the COI haplotypes was 0.003, ranging from 0.002 to 0.003. From 324 individuals, four unique mtDNA COII haplotypes (K1–K4, NCBI accession nos. PP056518-PP056521, Table S1) were identified. The haplotype content was 1.2% (4/324). Haplotype K1, prevalent across all the host populations, accounted for 95.37% of the individuals. Haplotype K3 was found in seven host populations, comprising 3.7% of the individuals. The remaining two haplotypes exhibited irregular distributions among different populations, with haplotype K2 detected in soybean and weed populations and haplotype K4 found in the peanut population. The mean genetic distance among the COII haplotypes was 0.004, ranging from 0.002 to 0.005.

Eleven unique mtDNA Cytb haplotypes (B1–B11, NCBI accession nos. PP056522-PP056532, Table S1) were identified from 310 individuals. The haplotype content was 3.5% (11/310). Haplotypes B1, B2, and B3 were present across all the surveyed hosts, representing 58.06%, 16.45%, and 13.55% of the individuals, respectively. Haplotype B4 was observed in nine host populations, accounting for 6.13% of the individuals. Haplotype B8 was found in six populations (3.23% of the individuals) and was consistently present in weed populations across both sampling years. The remaining infrequent haplotypes exhibited irregular distributions among different populations. The mean genetic distance among the Cytb haplotypes was 0.006, ranging from 0.002 to 0.012. In total, 21 combined haplotypes of COI, COII, and Cytb (COM1–COM21) were identified from 295 individuals. The haplotype content was 7.1% (21/295). Haplotypes COM1, COM2, and COM3 were present across all the surveyed hosts, representing 54.24%, 16.61%, and 10.17% of the individuals, respectively. The infrequent haplotypes COM16 and COM17 were detected in the soybean and weed populations. The mean genetic distance among the COM haplotypes was 0.002, ranging from 0.001 to 0.004.

**Figure 4:**
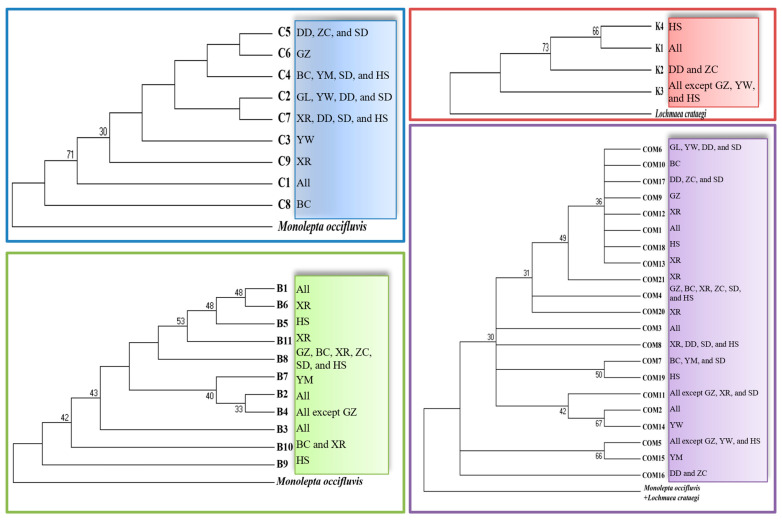
The phylogenetic relationships (NJ analysis) of leaf beetles were examined based on COI, COII, Cytb, and the combined haplotypes. The outgroup taxa were *Monolepta occifluvis* Gressitt and Kimoto (Coleoptera: Chrysomelidae) (Sequence ID: NC_045838.1) and *Lochmaea crataegi* (Forster) (Coleoptera: Chrysomelidae) (Sequence ID: OX387429.1). Bootstrap values were generated from 1000 replicates, and values <30% are not shown. COM1-COM21: the combined haplotype 1–21.

Cluster analysis of all the haplotypes did not reveal a clear host pattern (Figure 4). Many nodes were supported by low bootstrap confidence levels. All the haplotypes were distinct from the outgroup species. Haplotypes with shared variable sites formed strongly supported clades (e.g., the clade comprising COM2 and COM14 as well as the clade comprising COM5 and COM15). A clade containing haplotypes C2 and C7, each with a single variable site, exhibited a broader distribution. Infrequent haplotypes B5, B6, and B11 formed sister clades, and the three haplotypes had unique single variable sites. In a clade containing haplotypes B2 and B4, the two more widely distributed haplotypes showed similarities in variable sites. These results indicated a shared evolutionary and distributional pattern.

### 3.4. Genetic Diversity and AMOVA

The genetic diversity indices are shown in Table 3. Overall, the *Hd*, *K*, and *Pi* values of all the COI samples were 0.1412, 0.1456, and 0.0002, respectively. The haplotype range was 2–5, with a mean value of 2.9. The samples from the rice population had the most haplotypes. The mean *Hd* was 0.1641 (range: 0.0555–0.4727). The maize (L) population had the highest *Hd*, whereas the weed population had the lowest. Among the host populations, the average *K* value was 0.1703, ranging from 0.0555 to 0.5090. The *Pi* values based on host populations varied 0–0.0008, with an average value of 0.0002. The *Gst*, *Fst*, and *Nm* values were 0.0019, 0.0138, and 11.88, respectively. For the COII samples, the overall *Hd*, *K*, and *Pi* values were 0.0893, 0.1663, and 0.0003, respectively. The haplotype range was 1–3, with a mean value of 2. Samples from the soybean and weed populations had the most haplotypes, whereas those from the maize (L) and millet populations had the lowest. The mean *Hd* was 0.0827 (range: 0–0.1921). The sorghum populations had the highest *Hd*. Among the host populations, the average *K* value was 0.1552 (range: 0–0.3842). The *Pi* values based on the host populations were 0–0.0008, with an average value of 0.0003. The *Gst*, *Fst*, and *Nm* values were −0.0019, 0.0006, and 20.73, respectively.

For the Cytb samples, the overall *Hd*, *K*, and *Pi* values were 0.6144, 1.6229, and 0.0037, respectively. The haplotype range was 4–8, with a mean value of 5.2. Samples from the sunflower population had the most haplotypes. The mean *Hd* was 0.6026 (range: 0.4137–0.7468). The peanut population had the highest *Hd*, whereas the sorghum population had the lowest. Among the host populations, the average *K* value was 1.6109, with a range of 1.2315–1.8662. The *Pi* values based on the host populations varied 0.0028–0.0043, with an average value of 0.0037. The *Gst*, *Fst*, and *Nm* values were 0.013, 0.0006, and 13.56, respectively. For the combined fragment, the overall *Hd*, *K*, and *Pi* values were 0.6663, 1.9295, and 0.0013, respectively. The haplotype range was 5–10, with a mean value of 7.6. The mean *Hd* was 0.6717 (range: 0.4746–0.8). Among the host populations, the average *K* value was 1.9408, ranging from 1.4492 to 2.2909. The *Pi* values based on host populations varied 0.0009–0.0015, with an average value of 0.0013. The *Gst*, *Fst*, and *Nm* values were 0.008, −0.0019, and 14.2, respectively.

The AMOVA revealed that most of the total variation was within populations (Table 4). No clear pattern emerged based on genetic distance among the host populations (Table 5). Stable genetic distances were noted when comparing sorghum with the cabbage, sunflower, and peanut populations, and when comparing weed with the peanut, cabbage, sunflower, and maize populations. There was also stability when comparing maize with the millet, sorghum, sunflower, and soybean populations. The genetic distance between maize (L) and the other host populations for COI was found to be relatively large. This may be attributed to the small sample size of maize (L) populations. Additionally, differences in haplotype distribution among maize (Z) populations may contribute to this pattern. A similar phenomenon was observed in the millet and sorghum populations for COII and the peanut and sorghum populations for Cytb. No genetic distance was found between the soybean and weed populations.

## 4. Discussion

The leaf beetle *M. hieroglyphica* was present from July to October in 2022 and 2023, with daily catch numbers peaking from late July to mid-August over two successive years. This period coincided with the crucial growing season in Northeast China, consistent with previous reports [7]. The pest appeared earlier in maize, soybean, and weed hosts, persisting into later periods in maize, maize (L), and cabbage fields. No beetles were observed after crop harvest, suggesting a close relationship between their occurrence and plant growth. This result appears to be associated with the host transfer migration of some *M. hieroglyphica* individuals. Based on these observations, we propose a possible migration pathway: the leaf beetle initially appears in soybean and weed fields in early July, subsequently disperses to other crops, and eventually settles in cabbage and maize (L) fields by the later periods. The spatial dynamics and host transfer migration pathway align with previous reports [5]. Considering the insect’s lifespan, most host transfers may occur within a single step.

In terms of occurrence, the leaf beetle showed high numbers in maize and soybean fields. Given that maize and soybean are dominant crops in the region, and the pest’s occurrence area continues to expand [3], it is conceivable that its increasing numbers may result in considerable economic losses in the future. The highest number of beetles was found in the maize (L) field, indicating the insect’s preference for young leaves [6]. This finding mirrors observations in large farmland areas, where late-seeded maize is often severely damaged by the leaf beetle. A certain number of leaf beetles occurred in weed, sorghum, millet, and peanut fields, suggesting that these plants are suitable hosts. The low occurrence in sunflower fields could be explained by the widely spaced planting, whereas almost no individuals were observed in the wheat field, possibly due to earlier harvest times. Thus, it appears that wheat in the region is not affected by leaf beetles.

Li et al. [13] suggested that the genetic diversity of geographic populations in southern China was higher than that in northern China, possibly due to higher temperatures and more generations. Our research aimed to determine the level of genetic diversity among host populations. Genetic diversity is considered an important indicator of a species’ adaptive capacity in different environments [29,30,31,32,33,34,35,36,37,38]. Species with higher genetic diversity are expected to exhibit local adaptation and greater individual numbers [30,39]. However, there was no evidence to support a consistent relationship between genetic diversity and occurrence in the present study. For instance, high levels of genetic diversity were detected in the populations of maize, soybean, cabbage, and sunflower, but occurrence levels were not consistent among these hosts.

Different mtDNA fragments may evolve at different rates. More variable sites and haplotypes were found in the COI and Cytb fragments than in the COII fragment. Combining multiple mtDNA fragments can greatly increase the value of research [40,41,42]. Notably, the haplotype contents of *M. hieroglyphica* geographic populations previously reported in northern and southern China (2.9% and 5.7%, respectively) were higher than those in our study (1.2%) based on the same COII fragment [13,14]. It appears that the host populations exhibited a lower degree of haplotype content compared with geographic populations. Various haplotypes, including C1, K1, B1, B2, and B3, were found in all the host populations. These are ancestral haplotypes, the most frequent and widespread haplotype, which show robust adaptation to the local environment [39,40,43].

Based on the haplotype network and NJ tree, no distinct host pattern was formed. The haplotypes exhibited a weak host correlation, which was supported by other analyses. Estimates of the overall genetic differentiation coefficient (*Gst* and *Fst*) were low, and all *Nm* values exceeded 11, with *Nm* > 4 indicating strong gene flow in the analyzed populations [44,45]. The high level of gene flow was likely due to host transfer migration, which can prevent genetic divergence among host populations. Combined with the AMOVA, this analysis showed that most genetic variation was within populations. These findings do not strongly support the formation of host races. In contrast to previous studies on geographic populations, populations in northern and southern China exhibited similar levels of genetic divergence, with limited gene flow observed and some degree of variation among populations [13,14]. Therefore, based on the same COII fragment, host populations exhibited a higher degree of gene flow compared with geographic populations. This may be attributed to the limited flight capability of the studied species [6]; leaf beetles are not considered migratory, being capable of dispersal over short distances only.

Geographic isolation over extended periods can lead to genetic differentiation and the emergence of new subspecies [46,47,48,49]. Additionally, host specialization has been known to contribute to the formation of host races [50,51,52,53]. Although clear evidence for host-adapted races of the leaf beetle was not found in the current study, the data on haplotype distribution and genetic distances support some level of host genetic divergence. The evidence from haplotype distribution includes the following: (i) infrequent haplotypes, especially single haplotypes widespread among hosts; (ii) the consistent presence of infrequent haplotypes in the same hosts (C4 in maize and B8 in weeds) across both sampling years; and (iii) early occurrence of *M. hieroglyphica* populations in soybean and weed hosts, corresponding to the presence of specific infrequent haplotypes (e.g., C5 and K2) in these populations. These molecular data align with prior field observations. There was no evidence supporting genetic distance between the soybean and weed populations, although some degree of distance existed between the soybean and cabbage populations, likely due to differences in the occurrence period. The leaf beetle initially infests soybean and maize fields before appearing in cabbage and maize (L) fields, suggesting variance in genetic backgrounds associated with occurrence periods. Furthermore, results of genetic variation analysis coincided with the species’ morphological and biological characteristics. The peak periods of the leaf beetle differed among the host plant species, and there was a close correlation between emergence periods and body types [7]. Differences in life history and fecundity among host plants have also been reported [15]. Host divergence may contribute to these differences.

In recent years, leaf beetle damage to soybean, maize, and other crops has markedly increased in Northeast China. Our data on occurrence periods, occurrence levels, and genetic structures across the major crops in the region are crucial for developing effective pest control strategies. Although this study presents initial findings, its primary limitations include a relatively small genotyping sample size and a narrow geographical scope. To achieve more comprehensive results, further research should incorporate larger sample sizes and broader geographical ranges.

## Figures and Tables

**Table 1:** Information regarding the experimental site and molecular samples.

Population Code	Host	Sampling Methods			Molecular Samples	PCR-Positive Samples				Sampling Date		Harvest Date	
		Y	S	O		COI	COII	Cytb	COM	2022	2023	2022	2023
ZC	weed	-	✓	-	40	36	40	40	36	06-01~10-17	06-01~10-16	-	-
DD	soybean	✓	✓	-	40	38	39	35	35	06-01~10-17	06-01~10-16	09-25	09-22~09-30
BC	cabbage	✓	-	✓	30	29	30	30	29	09-08~10-17	08-22~10-16	10-10	10-01
YM	maize	✓	-	✓	40	38	40	39	38	06-01~10-17	06-01~10-16	10-12	10-07
YW	maize (L)	✓	-	✓	12	11	12	12	11	07-16~10-17	07-13~10-16	10-12	10-07
GZ	millet	✓	-	✓	30	25	30	28	25	06-01~10-17	06-01~10-16	10-04	10-11
GL	sorghum	✓	-	✓	30	29	29	29	29	06-01~10-17	06-01~10-16	10-04	10-06
XR	sunflower	✓	-	✓	30	29	30	30	29	06-01~10-17	06-01~10-16	08-15~09-30	08~18-09~25
HS	peanut	✓	-	✓	55	35	44	38	34	06-01~10-17	06-01~10-16	09-06~10-07	09~04-09~28
XM	wheat	✓	-	✓	0	0	0	0	0	06-01~07-17	06-01~07-16	07-20	07-19
SD	rice	✓	-	✓	30	29	30	29	29	06-01~10-17	06-01~10-16	10-02	10-03

Y: yellow traps; S: sweep sampling; O: visual observations; -: no data collected; ✓: this method was used; maize (L): late-cultivated maize.

**Table 2:** Primer information.

Gene	Primer Sequences	Primer Source
COI-F	AAAAATAGATTTTATCTAAGCCTTA	Designed from: NCBI MT178239
COI-R	TATGCTCGAGTATCTACATCTATAC	
COII-F	GAGCATCTCCTTTAATAGAACA	[13]
COII-R	GTATAAATGAGTGATTGGCTCC	
Cytb-F	AATTATGGWTGAYTAATTCGAAC	[13]
Cytb-R	AAATATCATTCAGGTTGAATATG	

**Table 3:** Leaf beetle genetic diversity among host populations.

Population Code	Number of Haplotypes (*H*)				Haplotype Diversity (*Hd*)				Average Number of Nucleotide Differences (*K*)				Nucleotide Diversity (*Pi*)			
	COI	COII	Cytb	COM	COI	COII	Cytb	COM	COI	COII	Cytb	COM	COI	COII	Cytb	COM
GZ	2	1	4	5	0.0800	0.0000	0.5158	0.6100	0.0800	0.0000	1.3836	1.5733	0.0001	0.0000	0.0032	0.0010
BC	3	2	6	8	0.1354	0.0666	0.6781	0.6970	0.1379	0.1333	1.7770	2.0098	0.0002	0.0003	0.0041	0.0013
GL	2	2	4	6	0.1330	0.1921	0.4137	0.5197	0.1330	0.3842	1.2315	1.7487	0.0002	0.0008	0.0028	0.0011
XR	3	2	8	10	0.1354	0.0666	0.7333	0.7635	0.1379	0.1333	1.6689	1.9064	0.0002	0.0003	0.0038	0.0012
YW	3	1	4	6	0.4727	0.0000	0.5606	0.8000	0.5090	0.0000	1.6818	2.2909	0.0008	0.0000	0.0039	0.0015
YM	2	2	5	7	0.1024	0.1423	0.6882	0.6899	0.1024	0.2846	1.8461	2.1479	0.0001	0.0006	0.0042	0.0014
DD	4	3	4	9	0.1536	0.1484	0.5395	0.6201	0.1578	0.2456	1.6134	2.0571	0.0002	0.0005	0.0037	0.0013
ZC	2	3	5	8	0.0555	0.0987	0.4628	0.4746	0.0555	0.1474	1.2871	1.4492	0.0000	0.0003	0.0029	0.0009
SD	5	2	5	9	0.2610	0.0666	0.6871	0.7758	0.2758	0.1333	1.7536	2.1674	0.0004	0.0003	0.0040	0.0014
HS	3	2	7	8	0.1126	0.0454	0.7468	0.7664	0.1142	0.0909	1.8662	2.0570	0.0001	0.0002	0.0043	0.0013
Total	9	4	11	21	0.1412	0.0893	0.6144	0.6663	0.1456	0.1663	1.6229	1.9295	0.0002	0.0003	0.0037	0.0013

**Table 4:** Information regarding the AMOVA.

Source of Variation	Variance Components				Percentage of Variation			
	COI	COII	Cytb	COM	COI	COII	Cytb	COM
Among populations	0.00085	−0.00041	0.006	0.00377	1.17067	−0.4957	0.73946	0.39074
Within populations	0.07205	0.08352	0.80607	0.96140	98.82933	100.4957	99.26054	99.60926

**Table 5:** Genetic distances among leaf beetle host populations were analyzed using molecular markers: COI (below the diagonal, upper values), Cytb (below the diagonal, lower values), COII (above the diagonal, upper values), and the combined fragment (above the diagonal, lower values).

	GZ	BC	GL	XR	YW	YM	DD	ZC	SD	HS
GZ		0.00000 0.00000	0.07949 0.00000	0.00000 0.00000	0.00000 0.00000	0.03975 0.01019	0.03187 0.00000	0.00779 0.00000	0.00000 0.00000	0.00000 0.01960
BC	0.00000 0.00000		0.00441 0.01550	0.00000 0.00000	0.00000 0.00000	0.00000 0.00000	0.00000 0.00000	0.00000 0.01543	0.00000 0.00000	0.00000 0.00000
GL	0.02132 0.00000	0.01818 0.01981		0.00441 0.01496	0.01868 0.00000	0.00000 0.05745	0.00000 0.00000	0.00683 0.00000	0.00441 0.05175	0.05724 0.08945
XR	0.00000 0.00000	0.00000 0.00000	0.01818 0.02218		0.00000 0.00000	0.00000 0.00000	0.00000 0.00000	0.00000 0.01217	0.00000 0.00000	0.00000 0.00186
YW	0.14270 0.00000	0.12279 0.00000	0.02783 0.00000	0.12279 0.00000		0.00000 0.00000	0.00000 0.00000	0.00000 0.00000	0.00000 0.00000	0.00000 0.00000
YM	0.01353 0.04262	0.00000 0.00000	0.03471 0.08112	0.01333 0.01104	0.17475 0.00000		0.00000 0.00000	0.00000 0.06583	0.00000 0.00000	0.02462 0.00000
DD	0.00000 0.00000	0.00000 0.00000	0.00000 0.00000	0.00000 0.00000	0.08682 0.00000	0.01093 0.00690		0.00000 0.00000	0.00000 0.00000	0.01303 0.01555
ZC	0.00232 0.00000	0.00304 0.01335	0.03031 0.00000	0.00304 0.01509	0.20990 0.00000	0.01743 0.07804	0.00000 0.00000		0.00000 0.05830	0.00000 0.09312
SD	0.00000 0.02277	0.00000 0.00000	0.00000 0.06908	0.00000 0.00000	0.03676 0.00000	0.00000 0.00000	0.00000 0.00000	0.00000 0.06218		0.00000 0.00000
HS	0.00000 0.04104	0.00000 0.00000	0.02053 0.08785	0.00000 0.00000	0.15081 0.00000	0.00000 0.00000	0.00000 0.01361	0.00028 0.08216	0.00000 0.00000	

## Data Availability

The data presented in this study are contained within the article and Supplementary Materials. All of the sequence data were deposited in the NCBI Genbank database under accession numbers PP038011–PP038019 and PP056518–PP056532.

## Supplementary Materials

The following supporting information can be downloaded at: https://www.mdpi.com/article/10.3390/insects16060605/s1, Table S1: Haplotype information.
