# Supplementary material for: Occurrence and Genetic Variation of Monolepta hieroglyphica (Motschulsky, 1858) (Coleoptera: Chrysomelidae), a Newly Emerging Pest, Among Hosts in Northeast China
Source: Insects. 2025 Jun 8;16(6):605. doi: 10.3390/insects16060605 (PMC12193814; doi:10.3390/insects16060605)
Supplement: Supplementary file 1 [file insects-16-00605-s001.zip › insects-3507750-supplementary.pdf]

Table S1. Haplotype information

Haplotype	NCBI accession number	Sequence
C1	PP038011	GCATAAATTATTCCTAGTATTCCAAATGTTTCTTTTTTTCTTCTTTCTTGACTAACAATATGAGAAATTATTCCAAATCCTGG<br>TAAAATTAAAATATAAACTTCTGGGTGTCCAAAGAATCAAAATAAATGTTGATATAAAATTGGATCTCCACCTCCTGCTG<br>GGTCAAAAAATGAAGTATTTAAGTTTCGATCTGTTAATAATATTGTAATTGCTCCAGCTAATACTGGTAATGATAATAGAA<br>GTAAAATAGCTGTAATTATAACAGCTCAAACAAATAAAGGTATACGATCTATAGATATTCCTCTTGGACGTATATTAATA<br>ATAGTCGTAATAAAATTAATAGCTCCTAAAATTGAAGAAATACCTGCTAAATGAAGTCTAAAAATTGCTAAATCTACAGA<br>AGCTCCTCCATGAGCAATATTAGATGATAATGGAGGATAAACAGTTCATCCTGTTCCTGCTCCTCTTTCAACAATTCTTCTT<br>ATAATTAATAAAAATAATGATGGGGGCAACAATCAAAATCTTATATTATTTATTCGAGGAAAAGCTATATCAGGAGCTCC<br>AATTATTAATGGAACTAACCAATTTCCGAATCCACCAATTATAATTGGT
C2	PP038012	GCATAAATTATTCCTAGTATTCCAAATGTTTCTTTTTTTCTTCTTTCTTGACTAACAATATGAGAAATTATTCCAAATCCTGG<br>TAAAATTAAAATATAAACTTCTGGGTGTCCAAAGAATCAAAATAAATGTTGATATAAAATTGGATCTCCACCTCCTGCTG<br>GGTCAAAAAATGAAGTATTTAAGTTTCGATCTGTTAATAATATTGTAATTGCTCCAGCTAATACTGGTAATGATAATAGAA<br>GTAAAGTAGCTGTAATTATAACAGCTCAAACAAATAAAGGTATACGATCTATAGATATTCCTCTTGGACGTATATTAATA<br>ATAGTCGTAATAAAATTAATAGCTCCTAAAATTGAAGAAATACCTGCTAAATGAAGTCTAAAAATTGCTAAATCTACAGA<br>AGCTCCTCCATGAGCAATATTAGATGATAATGGAGGATAAACAGTTCATCCTGTTCCTGCTCCTCTTTCAACAATTCTTCTT<br>ATAATTAATAAAAATAATGATGGGGGCAACAATCAAAATCTTATATTATTTATTCGAGGAAAAGCTATATCAGGAGCTCC<br>AATTATTAATGGAACTAACCAATTTCCGAATCCACCAATTATAATTGGT
C3	PP038013	GCATAAATTATTCCTAGTATTCCAAATGTTTCTTTTTTTCTTCTTTCTTGACTAACAATATGAGAAATTATTCCAAATCCTGG<br>TAAAATTAAAATATAAACTTCTGGGTGTCCAAAGAATCAAAATAAATGTTGATATAAAATTGGATCTCCACCTCCTGCTG<br>GGTCAAAAAATGAAGTATTTAAGTTTCGATCTGTTAATAATATTGTAATTGCTCCAGCTAATACTGGTAATGATAATAGAA<br>GTAAAATAGCTGTAATTATAACAGCTCAAACAAATAAAGGTATACGATCTATAGATATTCCTCTTGGACGTATATTAATA<br>ATAGTCGTAATAAAATTAATAGCTCCTAAAATTGAAGAAATACCTGCTAAATGAAGTCTAAAAATTGCTAAATCTACAGA<br>AGCTCCTCCATGAGCAATATTAGATGATAATGGAGGATAAACAGTTCATCCTGTTCCTGCTCCTCTTTCAACAATTCTTCTT

		ATAATTAATAAAAATAATGATGGGGGCAACAATCAAAATCTTATATTATTTATTCGAGGAAAAGCTATATCAGGAGCTCT<br>AATTATTAATGGAACTAACCAATTTCCGAATCCACCAATTATAATTGGT
C4	PP038014	GCATAAATTATTCCTAGTATTCCAAATGTTTCTTTTTTTCTTCTTTCTTGACTAACAATATGAGAAATTATTCCAAATCCTGG<br>TAAAATTAAAATATAAACTTCTGGGTGTCCAAAGAATCAAAATAAATGTTGATATAAAATTGGATCTCCACCCCCTGCTG<br>GGTCAAAAAATGAAGTATTTAAGTTTCGATCTGTTAATAATATTGTAATTGCTCCAGCTAATACTGGTAATGATAATAGAA<br>GTAAAATAGCTGTAATTATAACAGCTCAAACAAATAAAGGTATACGATCTATAGATATTCCTCTTGGACGTATATTAATA<br>ATAGTCGTAATAAAATTAATAGCTCCTAAAATTGAAGAAATACCTGCTAAATGAAGTCTAAAAATTGCTAAATCTACAGA<br>AGCTCCTCCATGAGCAATATTAGATGATAATGGAGGATAAACAGTTCATCCTGTTCCTGCTCCTCTTTCAACAATTCTTCTT<br>ATAATTAATAAAAATAATGATGGGGGCAACAATCAAAATCTTATATTATTTATTCGAGGAAAAGCTATATCAGGAGCTCC<br>AATTATTAATGGAACTAACCAATTTCCGAATCCACCAATTATAATTGGT
C5	PP038015	GCATAAATTATTCCTAGTATTCCAAATGTTTCTTTTTTTCTTCTTTCTTGACTAACAATATGAGAAATTATTCCAAATCCTGG<br>TAAAATTAAAATATAAACTTCTGGGTGTCCAAAGAATCAAAATAAATGTTGATATAAAATTGGATCTCCACCTCCTGCTG<br>GGTCAAAAAATGAAGTATTTAAGTTTCGATCTGTTAATAATATTGTAATTGCTCCAGCTAATACTGGTAATGATAATAGAA<br>GTAAAATAGCTGTAATTATGACAGCTCAAACAAATAAAGGTATACGATCTATAGATATTCCTCTTGGACGTATATTAATA<br>ATAGTCGTAATAAAATTAATAGCTCCTAAAATTGAAGAAATACCTGCTAAATGAAGTCTAAAAATTGCTAAATCTACAGA<br>AGCTCCTCCATGAGCAATATTAGATGATAATGGAGGATAAACAGTTCATCCTGTTCCTGCTCCTCTTTCAACAATTCTTCTT<br>ATAATTAATAAAAATAATGATGGGGGCAACAATCAAAATCTTATATTATTTATTCGAGGAAAAGCTATATCAGGAGCTCC<br>AATTATTAATGGAACTAACCAATTTCCGAATCCACCAATTATAATTGGT
C6	PP038016	GCATAAATTATTCCTAGTATTCCAAATGTTTCTTTTTTTCTTCTTTCTTGACTAACAATATGAGAAATTATTCCAAATCCTGG<br>TAAAATTAAAATATAAACTTCAGGGTGTCCAAAGAATCAAAATAAATGTTGATATAAAATTGGATCTCCACCTCCTGCTG<br>GGTCAAAAAATGAAGTATTTAAGTTTCGATCTGTTAATAATATTGTAATTGCTCCAGCTAATACTGGTAATGATAATAGAA<br>GTAAAATAGCTGTAATTATAACAGCTCAAACAAATAAAGGTATACGATCTATAGATATTCCTCTTGGACGTATATTAATA<br>ATAGTCGTAATAAAATTAATAGCTCCTAAAATTGAAGAAATACCTGCTAAATGAAGTCTAAAAATTGCTAAATCTACAGA<br>AGCTCCTCCATGAGCAATATTAGATGATAATGGAGGATAAACAGTTCATCCTGTTCCTGCTCCTCTTTCAACAATTCTTCTT<br>ATAATTAATAAAAATAATGATGGGGGCAACAATCAAAATCTTATATTATTTATTCGAGGAAAAGCTATATCAGGAGCTCC<br>AATTATTAATGGAACTAACCAATTTCCGAATCCACCAATTATAATTGGT

C7	PP038017	GCATAAATTATTCCTAGTATTCCAAATGTTTCTTTTTTTCTTCTTTCTTGACTAACAATATGAGAAATTATTCCAAATCCTGG<br>TAAAATTAAAATATAAACTTCTGGGTGCCCAAAGAATCAAAATAAATGTTGATATAAAATTGGATCTCCACCTCCTGCTG<br>GGTCAAAAAATGAAGTATTTAAGTTTCGATCTGTTAATAATATTGTAATTGCTCCAGCTAATACTGGTAATGATAATAGAA<br>GTAAAATAGCTGTAATTATAACAGCTCAAACAAATAAAGGTATACGATCTATAGATATTCCTCTTGGACGTATATTAATA<br>ATAGTCGTAATAAAATTAATAGCTCCTAAAATTGAAGAAATACCTGCTAAATGAAGTCTAAAAATTGCTAAATCTACAGA<br>AGCTCCTCCATGAGCAATATTAGATGATAATGGAGGATAAACAGTTCATCCTGTTCCTGCTCCTCTTTCAACAATTCTTCTT<br>ATAATTAATAAAAATAATGATGGGGGCAACAATCAAAATCTTATATTATTTATTCGAGGAAAAGCTATATCAGGAGCTCC<br>AATTATTAATGGAACTAACCAATTTCCGAATCCACCAATTATAATTGGT
C8	PP038018	GCATAAATTATTCCTAGTATTCCAAATGTTTCTTTTTTTCTTCTTTCTTGACTAACAATATGAGAAATTATTCCAAATCCTGG<br>TAAAATTAAAATATAAACTTCTGGGTGTCCAAAGAATCAAAATAAATGTTGATATAAAATTGGATCTCCACCTCCTGCTG<br>GGTCAAAAAATGAAGTATTTAAGTTTCGATCTGTTAATAATATTGTAATTGCTCCAGCTAATACTGGTAATGATAATAGAA<br>GTAAAATAGCTGTAATTATAACAGCTCAAATAAATAAAGGTATACGATCTATAGATATTCCTCTTGGACGTATATTAATA<br>ATAGTCGTAATAAAATTAATAGCTCCTAAAATTGAAGAAATACCTGCTAAATGAAGTCTAAAAATTGCTAAATCTACAGA<br>AGCTCCTCCATGAGCAATATTAGATGATAATGGAGGATAAACAGTTCATCCTGTTCCTGCTCCTCTTTCAACAATTCTTCTT<br>ATAATTAATAAAAATAATGATGGGGGCAACAATCAAAATCTTATATTATTTATTCGAGGAAAAGCTATATCAGGAGCTCC<br>AATTATTAATGGAACTAACCAATTTCCGAATCCACCAATTATAATTGGT
C9	PP038019	GCATAAATTATTCCTAGTATTCCAAATGTTTCTTTTTTTCTTCTTTCTTGACTAACAATATGAGAAATTATTCCAAATCCTGG<br>TAAAATTAAAATATAAACTTCTGGGTGTCCAAAGAATCAAAATAAATGTTGATATAAAATTGGATCTCCACCTCCTGCTG<br>GGTCAAAAAATGAAGTATTTAAGTTTCGATCTGTTAATAATATTGTAATTGCTCCAGCTAATACTGGTAATGATAATAGAA<br>GTAAAATAGCTGTAATTATAACAGCTCAAACAAATAAAGGTATACGATCTATAGATATTCCTCTTGGACGTATATTAATA<br>ATAGTTGTAATAAAATTAATAGCTCCTAAAATTGAAGAAATACCTGCTAAATGAAGTCTAAAAATTGCTAAATCTACAGA<br>AGCTCCTCCATGAGCAATATTAGATGATAATGGAGGATAAACAGTTCATCCTGTTCCTGCTCCTCTTTCAACAATTCTTCTT<br>ATAATTAATAAAAATAATGATGGGGGCAACAATCAAAATCTTATATTATTTATTCGAGGAAAAGCTATATCAGGAGCTCC<br>AATTATTAATGGAACTAACCAATTTCCGAATCCACCAATTATAATTGGT
K1	PP056518	TCTTACATCGTTATTTACTTGAAGGTCAATTAATTGAACTAATTTGAACTATTTTACCAACTATTATTTTAATTTTTATTGCT<br>ATTCCTTCTCTTCGATTAATTTATATTTTAGATGAAATTAATAATCCAATAATTACATTAAAAACAATTGGACATCAATGAT

		ATTGATCATATGAATATTCTGACTTTAAAAAAATTGAATTTGATTCTTATATAATTCCAACTAATGAAATTAATAACTTTAA<br>TTTTCGTTTATTAGACGTAGATAATCGAGTTATTTTACCATTTGAATCAAATATTCGATTATTAGTAACTGCAGCTGATGTA<br>ATTCATTCTTGAACAATTCCTTCTTTAGGAGTAAAAATTGATGCTACTCCTGGCCGATTAAATCAAGTTAGATTTACTTTAA<br>ATCGATCAGGATTATTTTA
K2	PP056519	TCTTACATCGTTATTTACTTGAAGGTCAATTAATTGAACTAATTTGAACTATTTTACCAACTATTATTTTAATTTTTATTGCT<br>ATTCCTTCTCTTCGATTAATTTATATTTTAGATGAAATTAATAATCCAATAATTACATTAAAAACAATTGGACATCAATGAT<br>ATTGATCATATGAATATTCTGACTTTAAAAAAATTGAATTTGATTCTTATATAATTCCAACTAATGAAATTAATAACTTTAA<br>TTTTCGTTTATTAGATGTAGATAATCGAGTTATTTTACCATTTGAATCAAATATTCGATTATTAGTAACTGCAGCTGATGTA<br>ATTCATTCTTGAACAATTCCTTCTTTAGGAGTAAAAATTGATGCTACTCCTGGCCGATTAAATCAAGTTAGATTTACTTTAA<br>ATCGATCAGGATTATTTTA
K3	PP056520	TCTTACATCGTTATTTACTTGAAGGTCAATTAATTGAATTAATTTGAACTATTTTACCAACTATTATTTTAATTTTTATTGCT<br>ATTCCTTCTCTTCGATTAATTTATATTTTAGATGAAATTAATAATCCAATAATTACATTAAAAACAATTGGACATCAATGAT<br>ATTGATCATATGAATATTCTGACTTTAAAAAAATTGAATTTGATTCTTATATAATTCCAACTAATGAAATTAATAACTTTAA<br>TTTTCGTTTATTAGATGTAGATAATCGAGTTATTTTACCATTTGAATCAAATATTCGATTATTAGTAACTGCAGCTGATGTA<br>ATTCATTCTTGAACAATTCCTTCTTTAGGAGTAAAAATTGATGCTACTCCTGGCCGATTAAATCAAGTTAGATTTACTTTAA<br>ATCGATCAGGATTATTTTA
K4	PP056521	TCTTACATCGTTATTTACTTGAAGGTCAATTAATTGAACTAATTTGAACTATTTTACCAACTATTATTTTAATTTTTATTGCT<br>ATTCCTTCTCTTCGATTAATTTATATTTTAGATGAAATTAATAATCCAATAATTACATTAAAAACAATTGGACATCAATGAT<br>ATTGATCATACGAATATTCTGACTTTAAAAAAATTGAATTTGATTCTTATATAATTCCAACTAATGAAATTAATAACTTTA<br>ATTTTCGTTTATTAGATGTAGATAATCGAGTTATTTTACCATTTGAATCAAATATTCGATTATTAGTAACTGCAGCTGATGT<br>AATTCATTCTTGAACAATTCCTTCTTTAGGAGTAAAAATTGATGCTACTCCTGGCCGATTAAATCAAGTTAGATTTACTTTA<br>AATCGATCAGGATTATTTTA
B1	PP056522	CAAGTAAGTAAGGATTATGTAATGTTAAAAATACTAATAAAAATGTTATAATTAATGCTCCTAAGATATCTTTAAAAGTA<br>AAATAAGGATGAAATGGAATTTTATCAATATCTCTTTTTGTTCCAATTGGATTTCTTGAACCTGTTTGATGTAAATATAATA<br>AGTGAATAATTATTAAGGCAAATACAATAAATGGTAAAATGAAATGAAATGTAAAAAATCGAGTTAATGTAGCATTATC<br>AACAGCAAATCCTCCTCAGATTCATTGAACAAGTAAATTTCCTAAGTATGGAATTGCTGATAGTAAATTAGTAATTACTGT

		GGCTCCTCAAAATGATATTTGTCCTCAAGGAAGAACATATCCAAGAAAAGCTGTTGCTATTGTAATAAAAAAAATTGTTA<br>CTCCAATTATTCATGTTTCTATTATATT
B2	PP056523	CAAGTAAGTAAGGATTATGTAATGTTAAAAATATTAATAAAAATGTTATAATTAATGCTCTTAAGATATCTTTAAAAGTA<br>AAATAAGGATGAAATGGAATTTTATCAATATCTCTTTTTGTTCCAATTGGATTTCTTGAACCTGTTTGATGTAAATATAATA<br>AGTGAATAATTATTAAGGCAAATACAATAAATGGTAAAATGAAATGAAATGTAAAAAATCGAGTTAATGTAGCATTATC<br>AACAGCAAATCCTCCTCAAATTCATTGAACAAGTAAATTTCCTAAGTATGGAATTGCTGATAGTAAATTAGTAATTACTGT<br>AGCTCCTCAAAATGATATTTGTCCTCAAGGAAGAACATATCCAAGAAAAGCTGTTGCTATTGTAATAAAAAAAATTGTTA<br>CTCCAATTATTCATGTTTCTATTATATT
B3	PP056524	CAAGTAAGTAAGGATTATGTAATGTTAAAAATACTAATAAAAATGTTATAATTAATGCTCCTAAGATATCTTTAAAAGTA<br>AAATAAGGATGAAATGGAATTTTATCAATATCTCTTTTTGTTCCAATTGGATTTCTTGAACCTGTTTGATGTAAATATAATA<br>AGTGAATAATTATTAAGGCAAATACAATAAATGGTAAAATGAAATGAAATGTAAAAAATCGAGTTAATGTAGCATTATC<br>AACAGCAAATCCTCCTCAAATTCATTGAACAAGTAAATTTCCTAAGTATGGAATTGCTGATAGTAAATTAGTAATTACTGT<br>AGCTCCTCAAAATGATATTTGTCCTCAAGGAAGAACATATCCAAGAAAAGCTGTTGCTATTGTAATAAAAAAAATTGTTA<br>CTCCAATTATTCATGTTTCTATTATATT
B4	PP056525	CAAGTAAGTAAGGATTATGTAATGTTAAAAATACTAATAAAAATGTTATAATTAATGCTCTTAAGATATCTTTAAAAGTA<br>AAATAAGGATGAAATGGAATTTTATCAATATCTCTTTTTGTTCCAATTGGATTTCTTGAACCTGTTTGATGTAAATATAATA<br>AGTGAATAATTATTAAGGCAAATACAATAAATGGTAAAATGAAATGAAATGTAAAAAATCGAGTTAATGTAGCATTATC<br>AACAGCAAATCCTCCTCAAATTCATTGAACAAGTAAATTTCCTAAGTATGGAATTGCTGATAGTAAATTAGTAATTACTGT<br>AGCTCCTCAAAATGATATTTGTCCTCAAGGAAGAACATATCCAAGAAAAGCTGTTGCTATTGTAATAAAAAAAATTGTTA<br>CTCCAATTATTCATGTTTCTATTATATT
B5	PP056526	CAAGTAAGTAAGGATTATGTAATGTTAAAAATACTAATAAAAATGTTATAATTAATGCTCCTAAGATATCTTTAAAAGTA<br>AAATAAGGATGAAATGGAATTTTATCAATATCTCTTTTTGTTCCAATTGGATTTCTTGAACCTGTTTGATGTAAATATAATA<br>AGTGAATAATTATTAAGGCAAATACAATAAATGGTAAAATGAAATGAAATGTAAAAAATCGAGTTAATGTGGCATTATC<br>AACAGCAAATCCTCCTCAGATTCATTGAACAAGTAAATTTCCTAAGTATGGAATTGCTGATAGTAAATTAGTAATTACTGT<br>GGCTCCTCAAAATGATATTTGTCCTCAAGGAAGAACATATCCAAGAAAAGCTGTTGCTATTGTAATAAAAAAAATTGTTA<br>CTCCAATTATTCATGTTTCTATTATATT

B6	PP056527	CAAGTAAGTAAGGATTATGTAATGTTAAAAATACTAATAAAAATGTTATAATTAATGCTCCTAAGATATCTTTAAAAGTA<br>AAATAAGGATGAAATGGAATTTTATCAATTTCTCTTTTTGTTCCAATTGGATTTCTTGAACCTGTTTGATGTAAATATAATA<br>AGTGAATAATTATTAAGGCAAATACAATAAATGGTAAAATGAAATGAAATGTAAAAAATCGAGTTAATGTAGCATTATC<br>AACAGCAAATCCTCCTCAGATTCATTGAACAAGTAAATTTCCTAAGTATGGAATTGCTGATAGTAAATTAGTAATTACTGT<br>GGCTCCTCAAAATGATATTTGTCCTCAAGGAAGAACATATCCAAGAAAAGCTGTTGCTATTGTAATAAAAAAAATTGTTA<br>CTCCAATTATTCATGTTTCTATTATATT
B7	PP056528	CAAGTAAGTAAGGATTATGTAATGTTAAAAATACTAATAAAAATGTTATAATTAATGCTCTTAAGATATCTTTAAAAGTA<br>AAATAAGGATGAAATGGAATTTTATCAATATCTCTTTTTGTTCCAATTGGATTTCTTGAACCTGTTTGATGTAAATATAATA<br>AGTGAATAATTATTAAGGCAAATACAATAAATGGTAAAATGAAATGAAATGTAAAAAATCGAGTTAATGTAGCATTATC<br>AACAGCAAATCCTCCTCAAATTCATTGAACAAGTAAATTTCCTAAGTATGGAATTGCTGATAGTAAATTAGTAATTACTGT<br>GGCTCCTCAAAATGATATTTGTCCTCAAGGAAGAACATATCCAAGAAAAGCTGTTGCTATTGTAATAAAAAAAATTGTTA<br>CTCCAATTATTCATGTTTCTATTATATT
B8	PP056529	CAAGTAAGTAAGGATTATGTAATGTTAAAAATACTAATAAAAATGTTATAATTAATGCTCCTAAGATATCTTTAAAAGTA<br>AAATAAGGATGAAATGGAATTTTATCAATATCTCTTTTTGTTCCAATTGGATTTCTTGAACCTGTTTGATGTAAATATAATA<br>AGTGAATAATTATTAAGGCAAATACAATAAATGGTAAAATGAAATGAAATGTAAAAAATCGAGTTAATGTAGCATTATC<br>AACAGCAAATCCTCCTCAAATTCATTGAACAAGTAAATTTCCTAAGTATGGAATTGCTGATAGTAAATTAGTAATTACTGT<br>GGCTCCTCAAAATGATATTTGTCCTCAAGGAAGAACATATCCAAGAAAAGCTGTTGCTATTGTAATAAAAAAAATTGTTA<br>CTCCAATTATTCATGTTTCTATTATATT
B9	PP056530	CAAGTAAGTAAGGATTATGTAATGTTAAAAATACTAATAAAAATGTTATAATTAATGCTCCTAAGATATCTTTAAAAGTA<br>AAATAAGGATGAAATGGAATTTTATCAATATCTCTTTTTGTTCCAATTGGATTTCTTGAACCTGTTTGATGTAAATATAATA<br>AATGAATAATTATTAAGGCAAATACAATAAATGGTAAAATGAAATGAAATGTAAAAAATCGAGTTAATGTAGCATTATC<br>AACAGCAAATCCTCCTCAAATTCATTGAACAAGTAAATTTCCTAAGTATGGAATTGCTGATAGTAAATTAGTAATTACTGT<br>AGCTCCTCAAAATGATATTTGTCCTCAAGGAAGAACATATCCAAGAAAAGCTGTTGCTATTGTAATAAAAAAAATTGTTA<br>CTCCAATTATTCATGTTTCTATTATATT
B10	PP056531	CAAGTAAGTAAGGATTATGTAATGTTAAAAATACTAATAAAAATGTTATAATTAATGCTCCTAAGATATCTTTAAAAGTA<br>AAATAAGGATGAAATGGAATTTTATCAATATCTCTTTTTGTTCCAATTGGATTTCTTGAACCTGTTTGATGTAAATATAATA

		AATGAATAATTATTAAGGCAAATACAATAAATGGTAAAATGAAATGAAATGTAAAAAATCGAGTTAATGTAGCATTATC<br>AACAGCAAATCCTCCTCAAATTCATTGAACAAGTAAATTTCCTAAGTATGGAATTGCTGATAGTAAATTAGTAATTACTGT<br>GGCTCCTCAAAATGATATTTGTCCTCAAGGAAGAACATATCCAAGAAAAGCTGTTGCTATTGTAATAAAAAAAATTGTTA<br>CTCCAATTATTCATGTTTCTATTATATT
B11	PP056532	CAAGTAAGTAAGGATTATGTAATGTTAAAAATATTAATAAAAATGTTATAATTAATGCTCCTAAGATATCTTTAAAAGTA<br>AAATAAGGATGAAATGGAATTTTATCAATATCTCTTTTTGTTCCAATTGGATTTCTTGAACCTGTTTGATGTAAATATAATA<br>AGTGAATAATTATTAAGGCAAATACAATAAATGGTAAAATGAAATGAAATGTAAAAAATCGAGTTAATGTAGCATTATC<br>AACAGCAAATCCTCCTCAGATTCATTGAACAAGTAAATTTCCTAAGTATGGAATTGCTGATAGTAAATTAGTAATTACTGT<br>GGCTCCTCAAAATGATATTTGTCCTCAAGGAAGAACATATCCAAGAAAAGCTGTTGCTATTGTAATAAAAAAAATTGTTA<br>CTCCAATTATTCATGTTTCTATTATATT
